# Feasibility and Effectiveness of Telehealth Occupational Therapy Home Modification Interventions

**DOI:** 10.5195/ijt.2018.6244

**Published:** 2018-08-03

**Authors:** MARNIE RENDA, JENNIFER E. LAPE

**Affiliations:** 1 CHATHAM UNIVERSITY, PITTSBURGH, PA, USA; 2 REBUILD INDEPENDENCE LLC, CINCINNATI, OH, USA

**Keywords:** Feasibility study, Home modifications, Home safety, Occupational therapy, Telehealth, Telerehabilitation

## Abstract

Despite the effectiveness of occupational therapy home modification interventions, persons with disabilities may not receive them due to service delivery costs, limited number of therapists, and expansive geographic service areas. The need for occupational therapy home modification interventions will increase with the rising U.S. aging population, incidence of chronic illness, and shift toward community-based care. This study examined the feasibility of telehealth occupational therapy home modification interventions using participant owned smart phones, tablets, or computers. A pretest posttest design (n=4) demonstrated improvement in home safety and perception of performance of daily activities. Participants reported satisfaction with the mode of intervention citing ease of use and reduction in client and caregiver burden. Two key implementation challenges were (1) inconsistent quality of synchronous audio and video and (2) limited funding for home modification interventions. A large-scale telehealth occupational therapy home modification interventions pilot study is warranted.

When an individual has a disability, it can be difficult to move around the home or complete activities of daily living, such as bathing, dressing, cooking, or going to work. Home environmental barriers can lead to functional impairments and reduced safety (Stark, 2001), increasing the risk for falls and injuries (Stark, 2004). Home evaluations conducted by occupational therapists have been shown to reduce caregiver burden (Gitlin, Corcoran, Winter, Boyce, & Hauck, 2001), delay institutionalization (Mann, Ottenbacher, Fraas, Tomita, & Granger, 1999) reduce falls (Cummings et al., 1999), increase self-perception of performance (Petersson, Kottorp, Bergstrom, & Lilja, 2009; Stark, Landsbaum, Palmer, Somerville, & Morris, 2009), and increase acceptance of home modifications (Aplin, de Jonge, & Gustafsson, 2013). Home modification interventions include evaluation; identification, selection, and acquisition of products; referrals to funding and social services; identification and oversight of adaptions or remodeling of the home; and client and caregiver education.

There is a growing demand for occupational therapy home modification services in the United States due to the aging population (Ortman, Velkoff, & Hogan, 2014), rising incidence of chronic illness (Thorpe & Howard, 2006), and a shift toward providing community-based services to enable people to live at home instead of in a nursing home (Snyder & Rudowitz, 2016). It is projected that the number of persons over 65 years old will be 55 million in 2020, 76 million in 2030, and 88.5 million in 2050 (Ortman et al., 2014). Research indicates that of individuals 85 and older, 74% require assistance with at least one activity of daily living such as bathing, dressing, or toileting (Federal Interagency Forum on Aging-Related Statistics, 2016). With the increase in number of older adults and disability rates, there has been an increase in community-based care to enable these individuals to continue to live at home. This trend is often referred to as aging in place. A study conducted by AARP (2004) found that 87% of people age 65 or older wanted to stay in their current home as they age.

Unfortunately, the majority of homes in the U.S. are older (U.S. Census Bureau, 2015) and have features that can be barriers for persons with disabilities. For example, older homes often have interior and exterior stairs, narrow hallways, poor lighting, low toilets, bathtubs, and showers with thresholds. Multiple studies have found that persons with mobility impairments have difficulty managing stairs, entrances, and moving between spaces indoors (Davis & Rodd, 2014; Hoenig et al., 2006; Iwarsson, Isacsson, & Lanke, 1998). Bathroom tasks are the most difficult for persons with disabilities and pose more safety concerns (Davis & Rodd, 2014; Gitlin, Miller, & Boyce, 1999; Hoenig et al., 2006; Mann et al., 1999). In fact, Gitlin et al., (1999) found that 79% of older adults had difficulty bathing, 62% had difficulty toileting, and 90% had difficulty during tub transfers. Therefore, it is anticipated that as the population continues to age, more people will encounter these challenges at home.

In addition to home environmental barriers, there are several healthcare system barriers impeding the delivery of needed on-site occupational therapy home modification interventions. These include limited funding for all components of home modification interventions, complex and lengthy approval processes, large geographic service areas, and limited skilled service providers. These barriers can restrict or preclude access to occupational therapy home modification interventions for persons in need. For example, an on-site occupational therapy home evaluation may be covered under healthcare insurance policies. However, there is no additional reimbursement for travel time to access clients in rural areas. As a result, people who qualify for services may not receive them or may experience significant service delivery delays.

After an evaluation is conducted, clients often discover that there is lack of funding to implement structural changes and acquire assistive technologies, or that the process of obtaining funding is long and complex. For example, common structural changes such as the installation of a ramp or grab bar are seldom, if ever covered by healthcare policies. Therefore, other funding sources such as waiver programs, non-profit organizations, or private funding are needed. A person with a disability may not be aware of the funding sources available nor have the ability to access them without assistance. Furthermore, due to the limited funding available, often multiple funding sources are required, making the process more complex.

Although assistive technologies such as walkers, wheelchairs, and hospital beds are covered by most health insurance policies, the approval process is long and cumbersome. For example, to obtain a power wheelchair, the person must be evaluated by a therapist, have a visit with a physician, and submit supporting medical records for approval. The duration of time between evaluation and acquisition of the wheelchair can be several months.

When a client is able to move forward with implementing structural changes or obtaining assistive devices, there are often no follow-up occupational therapy visits. The lack of follow-up visits reduces client satisfaction and can lead to twice as many negative outcomes (Aplin, de Jonge, & Gustafsson, 2015). Gitlin et al. (1999) found that after a single home visit, 80% of clients needed adjustments to assistive devices, 45% of clients needed additional equipment, and 65% of clients reported they were not using recommended equipment because they felt unsafe or the equipment fit poorly or malfunctioned.

Each of these barriers can prevent persons’ with disabilities from receiving needed home modification intervention services or can delay services for up to two years (Renda, 2016). Delays in home modifications result in diminished activities of daily living performance that may not improve when modifications are made (Petersson et al., 2009). Therefore, finding a means of providing effective, lower-cost and timely home modification interventions is essential.

Telehealth is a promising new service delivery model gaining momentum because it can improve access to care (Hoenig et al., 2006), reduce costs, and reduce wait times while maintaining patient satisfaction (Nakamura, Takano, & Akao, 1999). Over the past two decades, the body of evidence supporting remote home modification interventions has continued to grow. A systematic review, including four high-quality randomized control studies, found that clinical outcomes were equivalent for remote and in-person therapy visits (Steel, Cox, & Garry, 2011). Multiple feasibility and randomized control studies demonstrate that low-bandwidth technology, providing two-way audio and video communication, enables occupational therapists to provide effective remote home modification interventions (Dreyer et al., 2001, Hoenig et al, 2006; Hoffman, Russell, Thompson, Vincent, & Nelson, 2008; Nakamura et al., 1999; Sanford et al., 2006; Sanford et al., 2007).

The purpose of this study was to examine the feasibility and effectiveness of using a smartphone, tablet, or computer to deliver occupational therapy home modification interventions to improve (1) home safety and (2) perception of performance of daily activities in adults and older adults living at home with neuromuscular conditions.

## METHODS

A pretest-posttest design study was conducted over eight weeks by an occupational therapist (the first author) of Rebuild Independence LLC in Cincinnati, OH. Two commercially available standardized occupational therapy outcome measurement tools were administered before and after the home modification interventions to measure perception of occupational performance of client-identified daily activity problems and measured home safety. The study was approved by the Chatham University IRB.

## PARTICIPANTS

Prospective participants were referred by neurologists who learned about the study from an informational email and phone call. Participants and technology assistants completed verbal informed consent and were then screened to confirm inclusion criteria were met. Participant inclusion criteria were: age 18 years or older; difficulty completing one or more activities of daily living; access to a smart phone, tablet, or computer with camera; living in Ohio, Kentucky, or Indiana; English speaker; and had an individual to assist with positioning the identified electronic device in the home during sessions (referred to as a technology assistant throughout the article). Exclusion criterion was the presence of a medical power of attorney indicating cognitive deficits.

The participant-identified technology assistant inclusion criteria were: ability to hold and reposition the smartphone, tablet, or computer with verbal instructions; comfort using the smartphone, tablet or computer; ability to safely access all areas of the home; and familiarity with how the participant completes daily activities at home.

Eleven referrals were received. Five prospective participants met the criteria, and four completed the study. Participants included two males and two females ranging in age from 43 to 80 years old. All participants had a neuromuscular condition, used a mobility assistive device, and had a history of one or more falls within the past year. Refer to Table 1 for participant demographic information.

## IMPLEMENTATION

**Figure 1:**
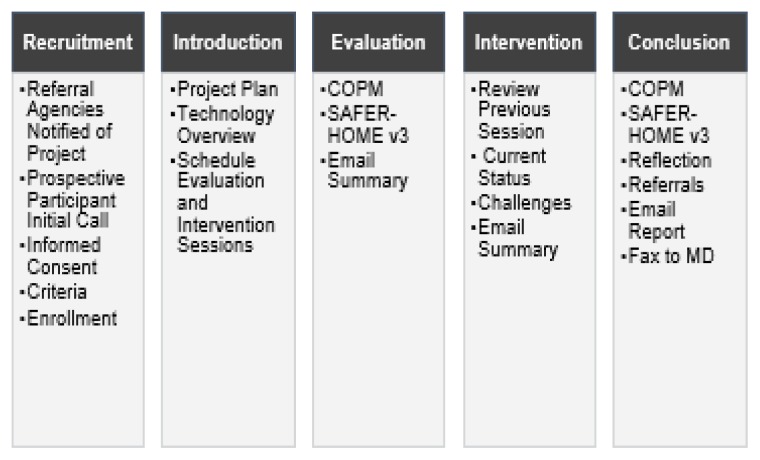
Phases of the study implementation. The figure illustrates the order of the study implementation phases, including a list of activities completed within each phase.

The study was implemented over an 8-week period and consisted of five phases: (1) recruitment, (2) introduction, (3) evaluation, (4), intervention, and (5) conclusion. Figure 1 provides an illustration of the study phases and activities completed during each phase. All interventions were conducted using the phone or the Doxy.me platform.

Participants and technology assistants were provided an overview of the study and a website with resources including: instructional videos on how to record the home and activity problems, possible funding sources, and educational handouts. During the evaluation phase, the occupational therapist (first author) used Doxy.me to administer the outcome measures and complete an occupational profile (values, interests, preferences, routines, rituals, experiences, supports, etc.). The technology assistants positioned the smart phone, tablet, or computer with instructions from the occupational therapist (first author). Due to poor video quality, some technology assistants were asked to email video recordings of the home and problem activities before the next session. The evaluation data was reviewed with the participant and used to collaboratively generate a list of client-centered occupational problems to address during the intervention phase.

The number of intervention sessions varied from two to six in order to address specific client needs. Additional visits were added to allow time to address problems identified after the evaluation, to schedule sessions with assistive technology vendors, and to provide additional time for participants to obtain and practice using assistive devices.

During the conclusion phase, the occupational therapist administered the outcome measures a second time and led a post-study participant and technology assistant reflection.

## OUTCOME MEASURES AND TECHNOLOGY

### COPM: PERCEPTION OF PERFORMANCE

The COPM is a client-centered outcome measure with strong reliability and validity that is administered using a semi-structured interview (Law et al., 2014). The COPM was used to identify occupational performance problems, and rate importance, perception of performance, and satisfaction on a scale of 1–10, with 1 being low and 10 being high.

### SAFER-HOME V.3: HOME SAFETY

The SAFER-HOME v.3 is a standardized outcome measure used to measure changes in home safety. Seventy-four home safety items were scored on a scale of 0–3: 0-no problem or not tested, 1-mild (1%–33%), 2-moderate (34–66%) and 3-severe (67%–100%) chance of a safety problem (Chiu et al., 2006). A decrease in the home safety score overtime indicates improvement in home safety.

### POST-INTERVENTION REFLECTION DISCUSSION

The occupational therapist (first author) led an informal post-intervention reflection discussion with participant and technology assistant pairs to gather qualitative data regarding their experiences participating in the study including: overall experience, suggested changes for future studies, and ease of use of technology.

### TECHNOLOGY

#### Doxy.me teleconferencing platform

The Doxy.me platform was used in this study because it meets HIPAA compliant standards, provides synchronous videoconferencing, and has secure document share, screen share, and screen capture picture features.

#### Participant technology

The devices use by the technology assistants during the study included: iPhone, Samsung Galaxy phone, Microsoft Surface, Dell computer, Samsung tablet, and iPad. The devices were owned by the technology assistants or participants and included access to internet services.

## RESULTS

### QUANTITATIVE OUTCOMES

#### PERCEPTION OF PERFORMANCE

**Figure 2:**
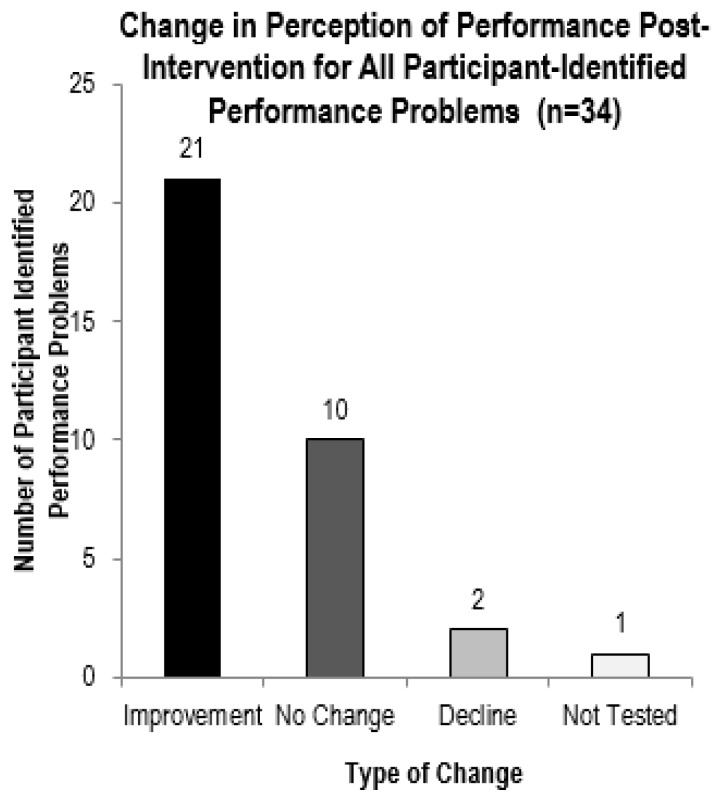
Change in perception of performance post-intervention for all participant-identified problems (n=34) using the COPM.

Each participant identified between seven and 13 occupational performance problems to address during the intervention phase. A combined total of 34 problems were identified amongst all participants. As illustrated in Figure 2, participants reported improvement in perception of performance in 21 of the problems, no change in 10, lower performance in two, and one problem was not tested post-intervention.

**Figure 3:**
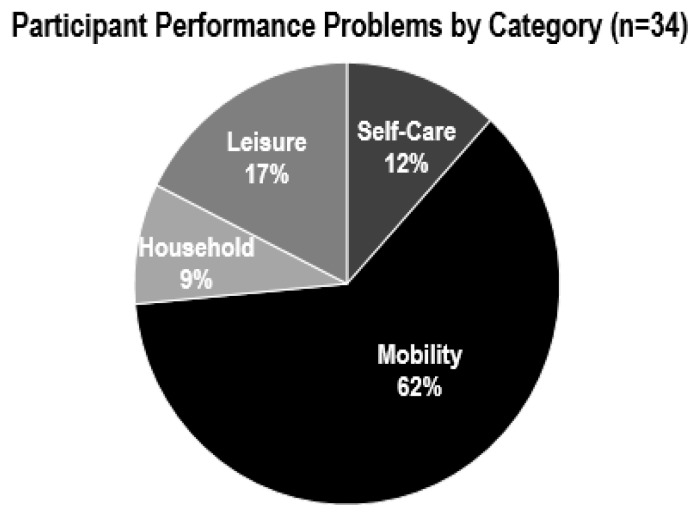
Comparison of participant-identified performance problems by category (n=34) using the COPM.

The percentage of problems with perceived improvement per participant are as follows: Participant A 100%, Participant B 60%, Participant C 38%, and Participant D 67%. The 34 problems were grouped into four categories: self-care, mobility, household, and leisure. The four self-care problems included: cutting food, dressing, showering, and medication. The problem categories are: self-care, mobility, household, and leisure management. The 21 mobility problems included: transfers, avoiding falls, stairs, accessing the community, and driving. The three household problems included: washing dishes, using the oven, and laundry. Finally, the six leisure problems included: walking a dog, drawing, visiting with friends, diagnosis peer support, gardening, and sitting in the front row in a van. Figure 3 illustrates the number of performance problems in each group.

**Figure 4:**
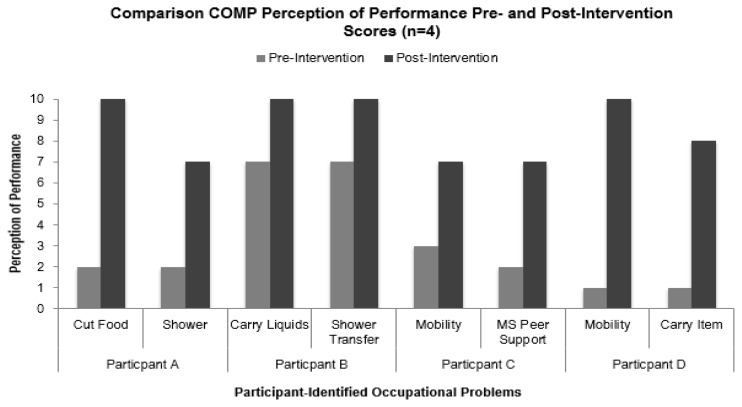
Comparison of pre and post-intervention perception of performance scores using the COPM (n=4). Two performance problems for each participant are included, along with pre- and post-intervention perception of performance scores

The post-intervention change in performance score ranged from −3 to 9. The average number of occupational problems with improved perception of performance was 5.25. The four occupational problems, with the greatest positive change in performance or satisfaction scores for each participant, are listed in Table 2. The table compares the pre- and post-intervention performance, satisfaction, and change scores for each of problem. Figure 4 compares the pre- and post-intervention perception of performance scores for each participant’s top two problems with the most improvement. The pre-intervention satisfaction scores ranged from 2 to 7 and the post-intervention satisfaction scores ranged from 0 to 9. The average change in satisfaction score was 5.2.

#### HOME SAFETY

Table 3 contains participant pre- and post-intervention SAFER-HOME v.3 scores and the change in home safety score. The largest score changes occurred in the following categories: household, kitchen, and environmental hazards. The average change score for all four participants was −20. A negative change in home safety scores indicates improved home safety.

### QUALITATIVE OUTCOMES

#### POST-STUDY REFLECTIONS

Participant and technology assistant responses were categorized into five themes: overall experience, benefits, caregiver burden, client-centered approach, and session number and length. Table 4 contains quotes categorized within each theme. All participants indicated that remote interventions were effective, addressed their individual needs, and eliminated the burden associated with leaving home for a healthcare appointment. All participants stated that they would either recommend telehealth visits to others or would participate in telehealth visits in the future. Participants A and B both stated that they achieved more in the remote visits than in previous in-person visits with occupational therapists.

#### TELEHEALTH TECHNOLOGY

High quality synchronous audio and video, using the Doxy.me platform, was inconsistent. After consultation with Doxy.me technology staff, it was determined that a large amount of documents on the occupational therapist’s (first author) MacBook Pro desktop was causing delays in streaming video and audio. After the documents were removed, the quality of the videoconferencing improved, but did not resolve completely. Due to these challenges, an asynchronous approach was added. Technology assistants were asked to record specific areas of the home and daily activities. The videos were transmitted via email or text message to the occupational therapist (first author) before the next intervention session. A text reminder with a link to the Doxy.me virtual office was sent to the technology assistant before each visit. Also, participants and technology assistants were emailed a summary of each session with a list of goals to complete before the next session.

## DISCUSSION

### PERCEPTION OF PERFORMANCE

**Figure 5:**
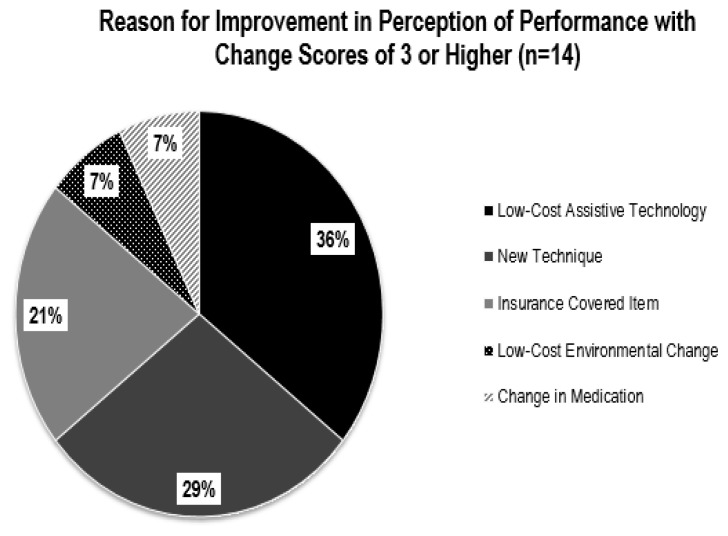
Reasons for improvement in perception of performance change scores of 3 or higher (n=14) using the COPM.

**Figure 6:**
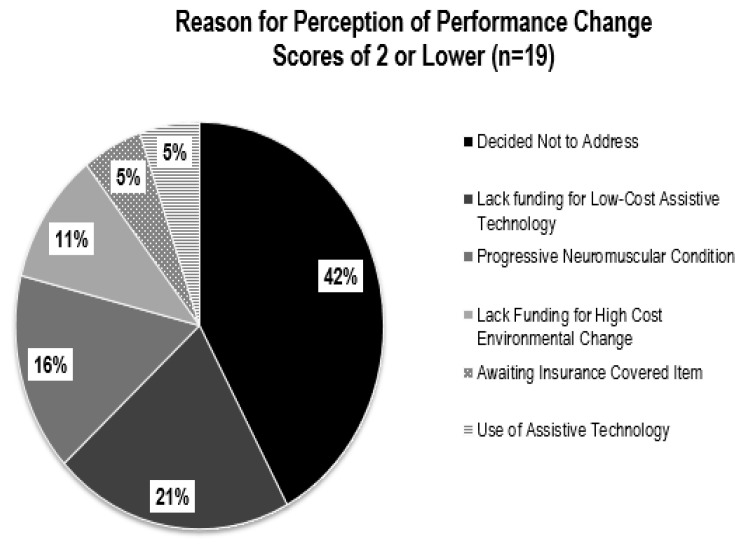
Reasons for perception of performance change scores of two or lower (n=19) using the COPM.

The degree of perception of performance change, as well as total number of problems addressed, varied between participants. The differences are likely due to the type and cost of the intervention, availability of funding, willingness to explore various solutions, and whether the home modification could be implemented within the study’s time frame. The most frequently identified problems were related to mobility. This is likely due to the participants’ diagnosis of neuromuscular conditions, use of mobility assistive devices, and history of falls. The reasons for improvement in perception of performance change scores of three or higher are illustrated in Figure 5 and the reasons for change scores of 2 or lower are provided in Figure 6.

Interventions consisting of low-cost assistive technologies or environmental changes paid for by the participant, mobility devices covered by insurance, or alterations in daily activity and mobility strategies, showed the greatest improvement in perception of performance scores. For example, perception of performance of cutting food increased by 8 points after Participant A purchased and began using an adaptive knife costing less than $10. Similarly, mobility improved 9 points after Participant D was fitted for and received a power wheelchair that was paid for by her healthcare insurance. Alternatively, problems that were most costly, or required more time to implement, showed the least amount of change in perception of performance. For example, the installation of a vertical platform lift, costing $15,000, required additional funding and building permits and therefore was not implemented in the study’s 8-week timeframe.

### HOME SAFETY

Participants’ improvement in home safety after occupational therapy home modification interventions is consistent with evidence found in the literature (Clemson, Mackenzie, Ballinger, Close, & Cummings, 2008; Cummings et al., 1999; Dreyer et al., 2001; Hoenig et al., 2006; Mann et al., 1999; Petersson, Lilja, Hammel, & Kottorp, 2008; Petersson et al., 2009; Sanford et al., 2004; Sanford et al., 2007; Sheffield, Smith, & Becker, 2012; Stark, 2004, Stark et al., 2009; Wilson et al., 2009). The areas of the home with the most safety concerns were household (cleaning the home and transporting food), bathroom and toileting (toilet and shower transfers), and mobility (managing stairs, chair and bed transfers and driving). Refer to Table 5 for a summary of the number of severe and moderate safety problems in each SAFER-HOME v.3 category. These results are consistent with previous studies that identified that bathroom tasks such as toilet transfers, shower transfers, and bathing are associated with safety concerns for persons with disabilities (Davis & Rodd, 2014; Gitlin et al., 1999; Hoenig et al., 2006; Mann et al., 1999).

### FEASIBILITY OF TELEHEALTH

The study successfully demonstrated that it is feasible to provide telehealth occupational therapy home modification interventions using a smart phone, tablet, or computer to improve home safety and perception of performance. The study results support the evidence that telehealth occupational therapy home modification interventions improve functional performance (as evidenced by improved home safety) and perception of performance of daily activities (Nakamura et al., 1999, Sanford et al., 2006).

Previous feasibility studies found that low bandwidth videoconferencing software could be used to administer telehealth occupational therapy evaluations and interventions using a technology staff member to manage the technology hardware and software (Dreyer et al., 2001, Hoenig et al, 2006; Hoffman et al., 2008; Nakamura, et al., 1999; Sanford et al., 2006; Sanford et al., 2007). In this study however, a combination of synchronous and asynchronous services was used due to technology limitations and the skill of the individual video recording the sessions. The audio and video processing speed was slow resulting in poor resolution at times. In addition, Doxy.me did not have a switch camera feature, which meant that technology assistants were unable to see what they were video recording during the sessions. Finally, unlike previous studies that used technology staff members, this study used untrained caregivers and friends using technology already available in the home. This resulted in several poor quality videos in which the participant was not centered in the frame, required additional instructions from the occupational therapist (first author), or required the technology assistants to record videos of the home environment and activities between sessions. Doxy.me launched a change camera feature during the final week of the study, which may significantly improve the ability of the occupational therapist to see the home environment and activities in future studies without the need of asynchronous home videos. Despite these challenges, all participants demonstrated improvements in home safety and perception of performance.

### PARTICIPANT EXPERIENCES

Participants and technology assistants were satisfied with the mode of service delivery, would recommend it to others, and would use it again in the future. The reduction in participant and caregiver burden was a common reason associated with satisfaction, as well as ease of use of Doxy.me, flexibility in scheduling sessions, and the client-centered approach.

### LIMITATIONS

Study limitations include: (1) a small sample size, (2) inconsistent videoconferencing quality during sessions, (3) funding for home modification interventions, (4) short study time frame to implement recommendations, and (5) potential researcher and participant bias. Four participants completed the study, limiting the generalizability of the results and ability to conduct statistical analysis on the data. While the participants found Doxy.me easy to use, the inconsistent videoconferencing quality disrupted sessions, resulting in the need for participants to record activities between sessions. The short time frame available to complete the home modification interventions did not provide the necessary time to access funding, acquire assistive technologies, alter home environments, or practice new techniques. Three to 12 months is a more realistic time frame to acquire funding, products, and make structural changes. As a result, several recommendations were not implemented, which may have negatively impacted home safety and perception of performance scores. Additionally, limited funding for telehealth occupational therapy home modification services impedes the provision of services outside of research studies. Finally, since the intervention, data collection, and analysis were conducted by the occupational therapist (first author), there could be researcher and respondent bias. The potential biases were limited by (1) the use of standardized outcomes measures and (2) review of data and outcomes by the second author.

## RECOMMENDATIONS

This feasibility study can be used as a foundation for the development of future studies examining the effectiveness of telehealth occupational therapy home modification interventions. A pilot study with a larger sample size is warranted. The larger sample size will aid in identifying specific populations and types of interventions that yield optimal outcomes. A potential alternative study design is to use existing home care providers such as case managers, nurses, and aides, as technology assistants. Using health care professionals, who are already going into the home and comfortable using technology, could eliminate the identified barriers of the inability of participants to find technology assistants and their discomfort using technology. Finally, further examination of the feasibility of using a smart phone, tablet, and computers across a wide variety of healthcare disciplines should be investigated.

## Figures and Tables

**Table 1:** Participant Demographics

Participant	Age	Sex	Primary Medical Condition	Frequency of Falls	Mobility Assistive Device	Living Environment	Technology Used	Technology Assistant
**A**	80	F	Progressive Supranuclear Palsy	Weekly	Multiple breaking system walker (U-Step Walker)	Ranch home with family in Ohio	-Microsoft Surface Pro -Dell Computer	Daughter
**B**	66	M	Parkinson’s Disease	Monthly	Multiple breaking system walker (U-Step Walker)	Second floor apartment in Ohio	-iPhone -iPad -Samsung Tablet	Friend
**C**	50	M	Multiple Sclerosis	Monthly	4 Wheeled-walker	2-Story home with wife in Kentucky	-iPhone	Wife
**D**	43	F	Multiple Sclerosis	Annually	Manual Wheelchair	Ranch home with husband and daughter in Ohio	-Samsung Phone -Samsung Tablet	Daughter

*Note.* Three out of four technology assistants used different devices during sessions. And, two of the technology assistants used two devices during a session: (1) to receive email links and documents and (2) to videoconference with the occupational therapist (first author).

**Table 2:** Comparison of Pre-and Post-Intervention COPM Performance and Satisfaction Scores

Participant	Occupational Problem	Performance			Satisfaction		
		Pre-test	Post-test	Change	Pre-test	Post-test	Change
***A***	Cut Food	2	10	8	2	10	8
	Shower	2	7	5	2	7	5
	Toilet Transfer	1	*9*	*8*	2	9	7
	Chair Transfer	1	6	5	1	5	4
	Dress Lower Body	1	7	6	1	3	2

***B***	Community Access	1	8	7	2	8	6
	Carry Liquids	7	10	3	6	10	4
	Shower Transfer	7	10	3	6	10	3
	Manage Medication	8	9	1	7	9	2

***C***	Bed Transfer	5	5	0	2	5	3
	Garage Stairs	4	5	1	1	6	5
	Mobility	3	7	4	1	5	4
	MS Peer Support	2	7	5	1	5	4

**D**	Dishes	6	7	1	2	9	7
	Mobility	1	10	9	1	10	9
	Carry Item	1	8	7	1	6	5
	Turn Stove Knobs	3	8	5	1	6	7

*Note.* The change score is the difference between the COPM pre and post-intervention scores. A positive change score indicates an improved perception of performance or satisfaction. Pre-Test= Pre-intervention score. Post-Test= Post-intervention score.

**Table 3:** Participant Change in SAFER-HOME v. 3 Home Safety Score

Participant	Pre-Intervention	Post-Intervention	Safety Score Change
**A**	43	22	−21
**B**	55	28	−27
**C**	48	28	−20
**D**	45	33	−12

*Note.* A negative safety score change indicates an improvement in home safety.

**Table 4:** Participant Post-intervention Reflection Quotes and Themes

Topic	Quote
Overall Experience	“This has been one of the more positive experiences that I have had with this whole Parkinson’s crap.” (Participant B). “I would recommend this to anyone in my situation.” (Participant B) “I feel more connected to the outside world now more than I did before I began working with you. Because you have allowed me to engage, you have brought clarity to some stuff” (Participant B). “It does make me feel less isolated.” (Participant B).
Benefits	“We go to a lot of appointments, lots of people come to the house, and you have dealt with more practical issues.” (Technology Assistant A) “It seemed like we were able to get more done because you said to me, OK that is your homework. So rather than that being the hour that you’re together- it just seems like a lot more was accomplished.” (Technology Assistant A) “You know where to get stuff, who to contact. It has made life a lot easier.” (Participant B) “It opened my eyes to see what might be done around the house. Because before I did not quite look at things the same. I could see what shouldn’t be, but not what should be.” (Technology Assistant B). “I was able to ask you questions. You would come back at me with answers, and I think it was great. ” (Participant C) I’m not good about writing things down, so it was nice to have the emails.” (Participant D)
Reduced Burden	“Usually, they want the client to do all of the running around and to get from point A to point B. And that is what is hard. You have eliminated that.” (Participant B). “I liked that I didn’t have to get dressed or clean my house.” (Participant D)
Ease of Use	“It was much easier.” (than going to an office) (Technology Assistant A). “I spoke with (Technology Assistant B) and we both agree that the way this has been set up is user friendly.” (Participant B). “It was easy to use.” (Participant C) “I thought it was kinda cool. I liked it.”(Participant D)
Length of Sessions	“I think it was just right for me.” (Participant A). “I thought I could use some more. I was surprised that it ended as fast as it did.” (Participant B).
Client-centered	“You got right to point of what her concerns were.” (Technology Assistant A) “There is no judgment or any of that, which is wonderful. You never did say, well you were supposed to get that done. You and I both know there are some folks like that… yeah….and that is when you dread it…because it’s like, oh man we are about to get busted because we didn’t get this done and … but I appreciate that you understand the goal is to help mom.” (Technology Assistant A).

*Note.* The author of the quote is listed in parenthesis immediately after the quote.

**Table 5:** SAFER HOME v.3 Categories with Largest Moderate and Sever Safety Concerns

SAFER-HOME Category	Severe Safety Concern	Moderate Safety Concern	Total
Household	1	6	7
Bathroom	1	8	9
Mobility	13	6	19

*Note.* The table represents the SAFER-HOME v. 3 categories with the highest number of severe and moderate safety concerns.
